# Isolated Unilateral Testicular Tuberculosis in a Young, Immunocompetent Patient: A Case Report

**DOI:** 10.7759/cureus.52902

**Published:** 2024-01-25

**Authors:** Jayanthi Gunasekaran, Rahul Ranjan, Ina Joshi, Priya Karlapudi, Rajiv M Gupta

**Affiliations:** 1 Department of Microbiology, Employees' State Insurance Corporation (ESIC) Medical College and Hospital, Faridabad, IND

**Keywords:** lj (lowenstein jensen) culture, ziehl-neelsen stain, rifampicin, lpa (line probe assay), cbnaat (cartridge based nucleic acid amplification), paucibacillary, testicular tuberculosis, extrapulmonary tuberculosis, genitourinary tuberculosis (gutb), tuberculosis

## Abstract

Testicular or epididymal tuberculosis is a rare form of extrapulmonary tuberculosis. Extrapulmonary tuberculosis of any form is very difficult to diagnose by microscopy because it is usually paucibacillary. Therefore, molecular methods play a major role in the diagnosis of extrapulmonary tuberculosis. We present a rare case of unilateral testicular tuberculosis in a 23-year-old immunocompetent patient with no history of contact with a known tuberculosis case. He presented to us with swelling on his testis for one month and a discharging sinus in the left testis for 15 days, along with an intermittent fever for a week. A pus swab from the discharging sinus of the testis was sent to microbiology, where a cartridge-based nucleic acid amplification test (CBNAAT) was done, which detected *Mycobacterium tuberculosis *complex (MTBC)*, *but resistance to rifampicin was not detected. A line probe assay was also done on the sample for first-line drugs, and no resistance was detected for rifampicin or isoniazid. The patient was started on first-line drugs in the intensive phase, and after the completion of two months of treatment, the patient’s discharge stopped and he showed clinical improvement. Being a young patient, if he had not been diagnosed and treated as early as possible, it could have led to infertility. This again emphasizes the importance of molecular methods for the diagnosis of extrapulmonary tuberculosis.

## Introduction

Extrapulmonary tuberculosis accounts for 15% of newly diagnosed cases of tuberculosis. The commonly affected areas of extrapulmonary tuberculosis include the lymph nodes, pleura, peritoneum, bones and joints, genitourinary system, and meninges [1].

Genitourinary tuberculosis (GUTB) represents a form of extrapulmonary tuberculosis that occurs in the kidneys, ureters, seminal vesicles, prostate, testis, vas deferens, and epididymis [2]. Genitourinary tuberculosis is a rare manifestation of extrapulmonary tuberculosis [3]. The most common anatomical sites affected are the prostate, epididymis, and seminal vesicles, with testicular tuberculosis accounting for 3% of cases [4].

Epididymal tuberculosis is known to occur in young adults. Epididymal tuberculosis causes extensive tissue damage and fibrosis of the epididymis and nearby genital tissues [5]. This can reduce the movement and quantity of sperm due to secondary atrophy and blockage of ducts, and as a result, fertility might be impaired [6].

## Case presentation

A 23-year-old male student from Haryana, India, weighing 58 kg, came with complaints of left scrotal swelling for one month, purulent discharge with a single sinus from the posterior surface of the scrotum for 15 days, and intermittent fever for one week. The patient was unmarried and denied having a sexual partner. The patient did not report any injury, discharge from the urethra, or previous surgery. The patient was empirically treated with doxycycline 100 mg twice a day (BD) in view of sexually transmitted diseases; however, the symptoms did not resolve. He was vaccinated with the bacillus Calmette-Guerin (BCG) vaccine. There was no significant history of contact with a known case of tuberculosis.

**Figure 1:**
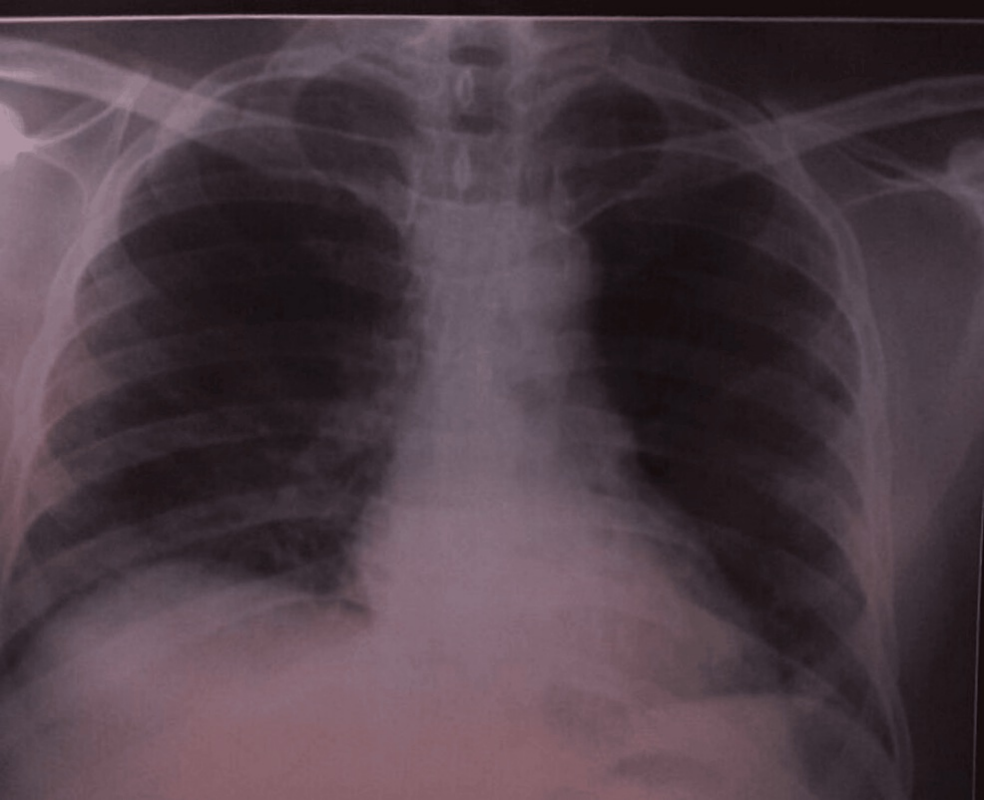
A chest X-ray (anteroposterior (AP) view) showing findings within normal limits

A year ago, the patient had a complaint of pain and swelling in the right wrist. He also had a history of trauma to the wrist. Further investigations were done to find the cause of the swelling. The total leucocyte count was 12890/ul, and the differential counts of neutrophils and monocytes were 66.6% and 8.3%, respectively. His C-reactive protein (CRP) was raised to 4.8 mg/dL, and his erythrocyte sedimentation rate (ESR) was 15 mm/hour. The rheumatoid factor (RA) was negative. Histopathology from the wrist showed mononuclear inflammatory infiltrates in the dermis. An MRI showed ill-defined enhancing soft tissue thickening in the volar aspect of the right wrist with bone marrow edema in all the carpal bones and the base of the second and third metacarpal bones (except pisiform), radiocarpal joint, and ulnar styloid process with cortical irregularity and marginal erosions suggestive of the infective etiology of tuberculosis/regional pain syndrome (given history of trauma). In Ziehl-Neelsen staining, acid-fast bacilli were not seen, and the cartridge-based nucleic acid amplification test (CBNAAT) did not detect *Mycobacterium tuberculosis* complex (MTBC) in the wrist biopsy sample. A differential diagnosis of right distal radioulnar joint instability due to triangular fibrocartilage complex injuries or tuberculosis was made. The patient was offered wristband support and was started on tab prednisolone 5 mg, tab sulfasalazine 500 mg, and tab etoricoxib 90 mg for three months. Anti-tubercular treatment was not started as there was no microbiological evidence. Other investigations were negative for hepatitis B surface antigen (HBsAg), hepatitis C antibodies, and HIV-1 and HIV-2 antibodies. The venereal disease research laboratory (VDRL) test was non-reactive. A chest X-ray was done, and the findings were not suggestive of pulmonary tuberculosis (Figure 1).

The wrist swelling clinically did not improve after the above treatment.

After one year of wrist swelling, the patient presented with the complaint of discharging sinuses from the left testis. On local examination, there was mild swelling of the left testis, and induration was felt on the left epididymis. A general systemic examination was normal. Investigations revealed CRP was raised to 4.8 mg/dL, the RA factor and anti-cyclic citrullinated peptide (anti-CCP) antibodies were negative, and ESR was 9 mm/hour. The antinuclear antibody (ANA) test was negative.

**Figure 2:**
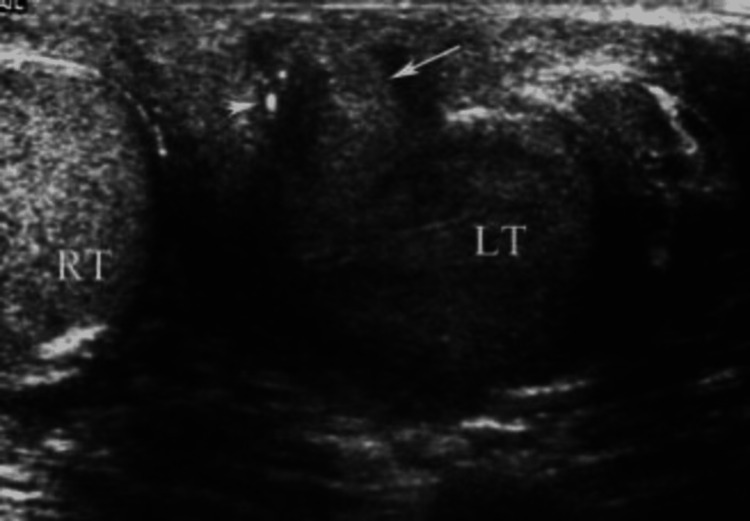
An ultrasound of the left testis shows a small sinus tract

The ultrasound of the inguinoscrotal region showed heterogeneous echotexture with an altered appearance of the left testis and epididymis and a small sinus tract extending to the cutaneous plane. The left epididymal head and body appeared bulky with increased vascularity, approximately 15 mm in diameter, suggesting left epididymitis. The right testis was found to be normal (Figure 2).

A differential diagnosis of left epididymo-orchitis/epididymal cyst was made. A swab was taken from the discharging sinus and sent for microbiological investigations like Gram staining, which did not show pus cells or microorganisms. Aerobic culture grew skin contaminants after 48 hours of aerobic incubation at 37 degrees Celsius.

**Figure 3:**
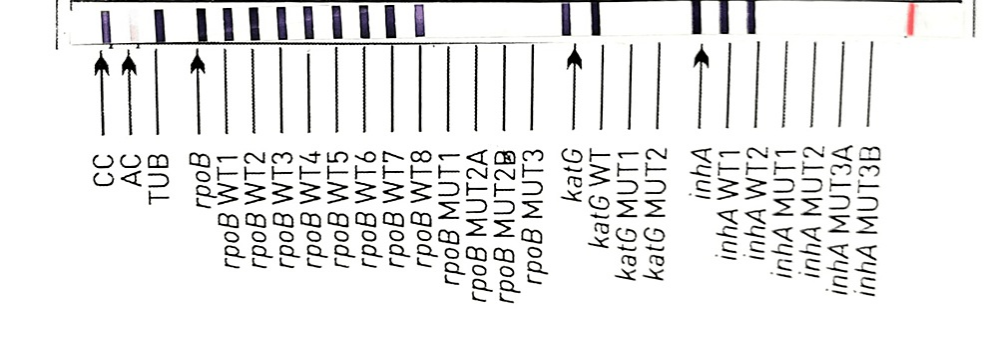
The line probe assay testing shows a sensitive pattern to rifampicin and isoniazid (absence of mutant bands and presence of all wild bands of the rpoB gene, katG gene, and inhA gene).

In the Ziehl-Neelsen stain, acid-fast bacilli were not seen. A CBNAAT was performed using GeneXpert (Ultra), in which MTBC was detected (trace) and rifampicin resistance was indeterminate. A line probe assay was performed on the sample using Genotype MTBDRPlus, which detected the MTBC and was susceptible to rifampicin and isoniazid (Figure 3).

The Lowenstein-Jensen culture didn't grow *Mycobacterium tuberculosis*.

Scrotal support was given to the patient, and he was referred to the directly observed treatment short (DOTS) course center for anti-tubercular treatment (ATT). In the intensive phase, four drugs were given orally, i.e., rifampicin (8-12 mg/kg/day), isoniazid (4-6 mg/kg/day), ethambutol (12-18 mg/kg/day), and pyrazinamide (20-30 mg/kg/day) for two months. At the end of the intensive phase, the patient clinically improved and the symptoms resolved, but the wrist swelling remained the same, so the etiology for wrist swelling might be unrelated to tuberculosis. The patient was further continued on the continuation phase for four months with the following drugs: rifampicin (8-12 mg/kg/day), isoniazid (4-6 mg/kg/day), and ethambutol (12-18 mg/kg/day) for the completion of treatment.

## Discussion

Genitourinary tuberculosis ranks third among the forms of extrapulmonary tuberculosis, with an estimated incidence of 2%-20% [7, 8]. Genital involvement occurs in 28% of patients with GUTB, although isolated testicular involvement is unusual. Differential diagnoses of testicular tuberculosis include testicular tumor, acute infection, infarction, and granulomatous infection [2]. The infection can reach any part of the male genital system through the bloodstream from the original site of the disease. Epididymitis is the most frequent type, which shows up as a lump in the scrotum or near the epididymis [9]. When an infection leads to inflammation and scarring, testicular tuberculosis can impair fertility. This is because the normal structure of the testicles is changed, and the ducts that carry sperm are blocked [10]. Reported cases of testicular tuberculosis in India are very few [4, 8]. This could be attributed to its typically paucibacillary nature, leading to potential oversight through conventional microscopy and culture methods.

We report a case of isolated testicular tuberculosis in a patient with no known risk factors other than being from a highly endemic country. His immune system was not compromised. He did not have HIV, diabetes, or any other serious health problems, nor was he on any long-term immunosuppressant drugs that could affect his immunity. Rarely, genital tuberculosis occurs alone. Contrary to what was observed in this case, it is more frequently an undesirable consequence of urinary tuberculosis [11]. The infection in this case may have resulted from the high prevalence of tuberculosis in India and the likely transmission of the bacteria through the bloodstream [8].

Despite clinical suspicion of tuberculosis during the patient's previous presentation with wrist swelling, microbiologically, tuberculosis was ruled out. However, in the second presentation of the testis, the diagnosis of tuberculosis was clearly established by molecular methods such as CBNAAT and line probe assays.

## Conclusions

Testicular tuberculosis is hard to diagnose correctly or on time because genital tuberculosis has vague symptoms and shows up late. There are fewer quick, accurate, and reliable tests for this condition. Therefore, to prevent effects like infertility, testicular tuberculosis should be diagnosed and treated at the early stage of the disease. Proper clinical insight and molecular diagnostic facilities are required to diagnose any form of extrapulmonary tuberculosis, as they are usually paucibacillary and might be missed if tested only by smear microscopy and conventional culture. Sometimes, tuberculous epididymitis is the only sign of GUTB. Therefore, even if there are no other symptoms or tests that show kidney or urinary tract tuberculosis, men who have epididymal lumps need to have a fine needle aspiration biopsy and a molecular test like CBNAAT. This will help to diagnose and treat the infection early and avoid complications.
